# Serum miR-125b levels associated with epithelial ovarian cancer (EOC) development and treatment responses

**DOI:** 10.1080/21655979.2020.1736755

**Published:** 2020-03-04

**Authors:** Zhonghua Chen, Xiaoli Guo, Shukai Sun, Caixia Lu, Liming Wang

**Affiliations:** a Department of Gynecology, The Affiliated Hospital of Qingdao University, Qingdao, Shandong, China; b Department of Obstetrics and Gynecology II, People’s Hospital of Gaotang County, Liaocheng, Shandong, China; c Department of Reproductive Medicine, The Affiliated Hospital of Qingdao University, Qingdao, Shandong, China; d Department of Clinical Lab, The Affiliated Hospital of Qingdao University, Qingdao, Shandong, China

**Keywords:** Epithelial ovarian cancer, Biomarkers, Diagnosis, Chemotherapy, Mir-125

## Abstract

Downexpression of miRs was associated with tumor development, progression, and metastasis. This study explored the serum levels of miR-125b in patients with epithelial ovarian cancer (EOC) and to assess its diagnostic value and monitor treatment responses for patients with EOC. A total of 379 individuals were recruited and assigned to the study groups. RT-qPCR analysis was performed to conﬁrm the association of serum miR-125b levels with tumor stages and treatment responses. The median serum levels of miR-125b in patients with EOC were signiﬁcantly lower than that of other controls (P < 0.0001). Serum miR-125b in patients with high FIGO stage (III+IV), lymph node metastasis, and chemoresistance were lower than that in patients with early-stage (stage I+ II; P < 0.001), without lymph metastasis (p = 0.032) and chemosensitivity (P < 0.001). Low levels of miR-125b had a poor prognosis in patients with EOC. Using a median value of 0.748 to separate EOC from other controls, the sensitivity and speciﬁcity reached 0.76 (95% CI 0.75 to 0.85) and 0.416 (95% CI 0.26 to 0.55), respectively. Serum miR-125b showed a statistically signiﬁcant difference between preoperative and postoperative patients in surgical patient groups (P = 0.003). Serum miR-125b levels were lower in patients with chemoresistance than that in patients with chemosensitivity (P < 0.0001). Serum miR-125b in combination with serum CA125 improved both sensitivity and speciﬁcity in diagnosis of EOC (P < 0.001). This study demonstrated that serum miR-125b levels were a useful diagnostic biomarker and biomarker to predict the responses to chemotherapy in patients with EOC.

## Introduction

Epithelial ovarian cancer (EOC) comprises 90% of all forms of ovarian cancer (OC) cases and is characterized by heterogeneity at histopathological, clinical, and molecular level [1]. With advances in diagnosis and treatment, 5-year survival has improved significantly over the last three decades, but the overall cure rate remains at 30% [2].

The exact cause for the ovarian malignancy still remains unknown. Poor outcomes relate to late diagnosis and persistence of dormant, drug-resistant cancer cells [3]. EOC is usually diagnosed at the advanced stages of disease, also frequently metastasizes to the lymph nodes and distantly, which makes curable surgery impossible and also makes chemotherapy and radiotherapy ineffective. Therefore, to improve the outcome of these patients, noninvasive and rapid diagnostic methods with high sensitivity and speciﬁcity are urgently needed. As such, both scientiﬁc and clinical researchers put most of their efforts into discovery of novel biomarkers and molecular targets.

MicroRNAs (miRNAs) are small non-coding RNAs of 19–25 nucleotides that can modulate gene expression by hybridizing to complementary target mRNAs, resulting in either mRNA degradation or direct inhibition of translation [4]. miRNA can also activate gene expression by interacting with complementary regions found in the promoter and coding region, as well as the 3ʹUTR of mRNA targets [5]. Expressed miRNAs provide a novel layer of regulation for human gene expression and play important roles in diverse biological processes [6]. Alterations in miRNAs have been detected in human ovarian cancers [7]. The clinical importance of miRNAs has been demonstrated for several types of cancer, including ovarian cancer [8]. The tendency is to determine the extracellular miRNAs that are present in various body ﬂuids, which are stable because they are packed in extracellular vesicles [9]. Free circulating miRNAs are considered to be valuable biomarkers in multiple pathologies. After their biogenesis, miRNAs are secreted from cells, and they can be found in a variety of body ﬂuids, such as serum/serum, saliva, urine, breast milk, cerebrospinal ﬂuid, ascites, pleural eﬀusion, and vaginal discharge [10–12]. There is no doubt that circulating cell-free miRNA are considered to be clinically relevant for ovarian cancer diagnosis, prognosis, and therapeutics [13]. Multiple miRNAs have been considered as potential clinical cancer biomarkers, particularly in ovarian cancer, and their expression proﬁles have been analyzed in ovarian cancer cell lines, in xenograft mice models for EOC, in surgically excised specimens from EOC patients, or in the serum/serum of EOC patients [10,13].

The miR-125 family members (miR-125a and miR-125b) have been reported to regulate tumor cell proliferation and metastasis. The family members are present in the mammalian genome: miR-125a localizes to chromosome 19q13, while miR-125b localizes to chromosome 11q23. Current studies revealed that dysregulation of miR-125a and miR-125b can lead to disease pathogenesis and tumorigenesis [14]. It has recently found that miR-125b expression was markedly lower in the EOC specimens. Ectopic expression of miR-125b in EOC cells significantly inhibited tumor invasion, proliferation, and colony formation [15,16], suggesting that miR-125b is an inhibitor. A previous study showed that levels of serum miR-125b in patients with cancer were significantly associated with cancer progression and metastasis. This indicates that miR-125b was an independent marker for tumor diagnosis and prognosis [17]. However, to the best of our knowledge, there is currently no single study with a large clinical sample size reporting diagnostic and prognostic signiﬁcance of miR-125b levels in ovarian cancer.

In this study, 394 cases diagnosed with either EOC or healthy controls, or benign ovarian disease were recruited. It aims to investigate whether serum levels of miR-125b could be a useful biomarker for diagnosis and prediction of treatment response in patients with EOC.

## Materials and methods

### Patients and serum samples

A total of 42 healthy controls, 30 benign tumors (21 serous and 10 mucinous), 35 borderline tumors (16 serous, 14 mucinous, 2 clear cell, and 3 mixed), 152 epithelial ovarian cancers (103 serous, 12 mucinous, 20 endometrioid, 9 clear cell, 6 transitional cell, and 2 mixed) were selected from patients who enrolled in the Affiliated Hospital of Qingdao University between February 2010 and October 2014. EOC was staged according to the International Federation of Gynecology and Obstetrics (FIGO) staging system and graded according to the WHO grading system. The age of patients ranged from 23 to 78 years (average 50.8 years). Serum (EDTA-K2 anticoagulant) samples from all participants were collected and then stored them at −20°C until use.

A total of 120 consecutive patients with newly diagnosed EOC were also selected, of the patients, 47 patients received surgery and 83 patients underwent chemotherapy. For surgical patients, serum samples were collected 3 days before surgery and 3 to 7 days after surgery. For chemotherapy patients, who were treated with platinum-taxane regimen, six standard courses of carboplatin 5–7.5 area under the curve (AUC) and paclitaxel 175 mg/m^2^ and modified according to the patient’s general status, was introduced in all cases. Platinum-sensitive tumors were identified when there was no relapse ≥6 months following completion of the chemotherapy. Resistant patients were defined as patients with primary chemo-refractory tumors (progression despite treatment with a first‑line chemotherapy). Platinum-resistance was also diagnosed when relapse occurred ≤6 months following the completion of chemotherapy. The sensitive patients to chemotherapy treatment were 48 cases, and 35 patients were resistant to chemotherapy treatment.

### Serum preparation, RNA isolation, and quantitative reverse transcription–polymerase chain reaction (RT-PCR)

To obtain serum samples, 10 mL of peripheral blood was drawn into separate gel tubes and then subjected within 30 min to centrifugation at 1,500 *g* for 10 min at 4°C. The supernatants were transferred to 1.5-mL tubes and stored at −80°C until use. To detect miRN-125b expression, 600 μL of the serum sample from each participant was subjected to RNA isolation using a mirVana PARIS RNA isolation kit (Applied Biosystems, Foster City, CA, USA), according to the manufacturer’s protocol. The RNA concentration was determined using a NanoDrop ND-1000 spectrophotometer (NanoDrop Technologies) and a 15% denatured polyacrylamide gel. The RNA samples were subjected to a reverse transcription reaction using a TaqMan MicroRNA Reverse Transcription Kit (Applied Biosystems), according to the manufacturer’s instructions. Subsequently, qPCR was carried out on the serum samples in triplicate using TaqMan 2× Universal PCR Master Mix with no AmpErase UNG (Applied Biosystems) on an ABI 7500 real-time PCR system (Applied Biosystems). The primers used for cloning of miRNAs were miR-125b, 5ʹ-CCCCCGCTAGCTCTT-GTTTTGCTTTGCTTTGTC-3ʹ, 5ʹ-CCCGAATTCA-CCAAATTTCCAGGATGCAA-3ʹ. The control primers (U6) were 5ʹ-CTCGCTTCGGCAGCACA-3ʹ, 5ʹ-AACGGTTCACGAATTTGCGT-3ʹ. qPCR amplification conditions were set to an initial cycle of 95°C for 10 min followed by 40 cycles of 95°C for 15 s and 60°C for 1 min. The cycle threshold (Ct) values were calculated using SDS 2.0.1 software (Applied Biosystems). No template controls were used in either the RT or PCR steps to ensure target-specific amplification. The control miRNA was U6 small nuclear RNA (snRNA), according to a previous study. The average expression levels of serum miRNA were calculated using the 2^−ΔCt^ method relative to the average of U6 snRNA (U6).

## Detection of tumor marker CA125

The serum levels of CA125 were also measured using a commercially available ELISA kit (R&D Systems), according to the manufacturer’s protocols.

### Statistical analysis

Statistical analyses were performed using SAS9.2 software (SAS Institute Inc.). The data were summarized as median. Cutoff values of miR-125b expression were taken as the medians of expression levels in serum from the patients. Differences (if any) were evaluated using the *t*-test or the rank-sum test or Student’s *t*-test. To assess the diagnostic performance of miR-125b and miR-125b in combination with CA125 between patients with EOC and non-EOC, the models were assessed by logistic regression. Receiver operating characteristic (ROC) curves were generated to compare the predictive sensitivity, speciﬁcity, and area under the curve (AUC) with 95% CI. Kaplan–Meier survival curves were used to evaluate the association between the expression levels of miR-125b and patient survival rate. The differences between the studied groups were determined by using the Mann–Whitney U test or Kruskal–Wallis. Spearman’s rank correlation coefficient was used in order to determine the statistical dependence between two variables. Multivariate analysis was used to estimate correlations between ≥3 variables. P < 0.05 was considered to indicate a statistically significant difference.

## Results

### qRT-PCR analyses for serum miR-125b

**Figure 1.**
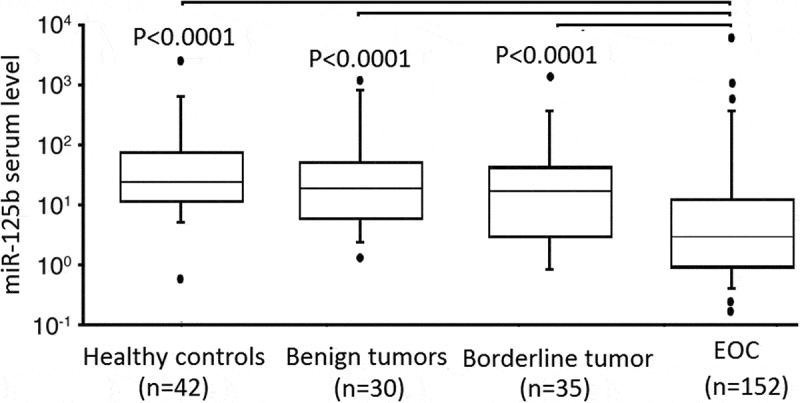
RT-qPCR detection of serum levels of miR-125b in healthy control, patients with EOC and controls.

The serum miR-125b levels in 42 healthy controls, 40 benign tumors, 35 borderline tumors, and 162 EOC were selected and assessed. The data showed that serum miR-125b levels were signiﬁcantly lower in patients with EOC than those of others (P < 0.0001; Figure 1). No significant difference was showed between patients with benign tumors and borderline tumors with healthy controls (P > 0.05, respectively).

### miR-125b is a marker for predicting prognosis and diagnosis of EOC

**Figure 2.**
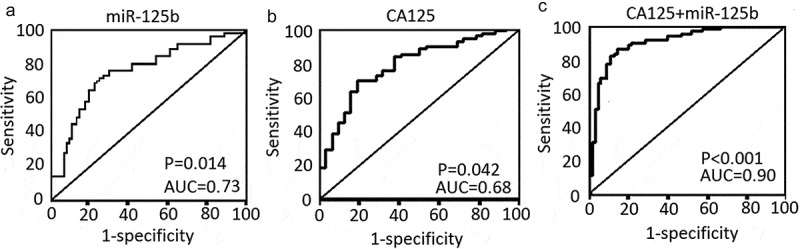
The levels and diagnostic values of CA125 and miR-125b in patients with EOC. (a) The sensitivity and the speciﬁcity of miR-125b levels in diagnosis of EOC. (b) The sensitivity and the speciﬁcity of CA125 levels in diagnosis of EOC. (c) The sensitivity and the speciﬁcity of CA125 + miR-125b levels in diagnosis of EOC.

qRT-PCR analysis showed that serum miR-125b levels were significantly lower in patients with high FIGO stage (III+IV) and lymph node metastasis than that in patients with low FIGO stage (I/II+IV) (P < 0.001) and without lymph node metastasis (P = 0.032). However, there was no signiﬁcant difference between patients with different ages, histological subtypes, tumor grade, distant metastasis, and CA125 levels, respectively (Table 1) (P > 0.05). ROC curve analyses were performed to evaluate the predictive power of miR-125b for EOC and illustrated in Figure 2(a). The serum miR-125b levels could discriminate patients with EOC from other controls, with the sensitivity and speciﬁcity reached 0.76 (95% CI 0.75 to 0.85) and 0.416 (95% CI 0.26 to 0.55) from a cutoff score of 0.748 (P = 0.014).

**Table 1.** Association of miR-125b level expression with clinicopathological characteristics in patients with ovarian cancer.

Groups	No.	Relative miR-125b level (fold)	*p*-Value
Age			0.274
≤50	70	0.836 ± 0.147	
<50	82	0.897 ± 0.153	
Histological subtype			0.073
Serous	103	0.812 ± 0.136	
Others	49	0.856 ± 0.145	
FIGO stage			<0.001
I/II	30	0.924 ± 0.158	
III/IV	122	0.682 ± 0.132	
Tumor grade			0.186
Well + moderate	70	0.846 ± 0.162	
Poor	82	0.836 ± 0.247	
CA125			0.242
≦35	23	0.826 ± 0.116	
>35	129	0.848 ± 0.132	
Lymph node status			0.032
No	103	0.875 ± 0.158	
Yes	49	0.804 ± 0.162	
Distant metastasis			0.146
No	127	0.868 ± 0.125	
Yes	25	0.829 ± 0.144

### Serum miR-125b levels with surgery or chemotherapy in patients with EOC

**Figure 3.**
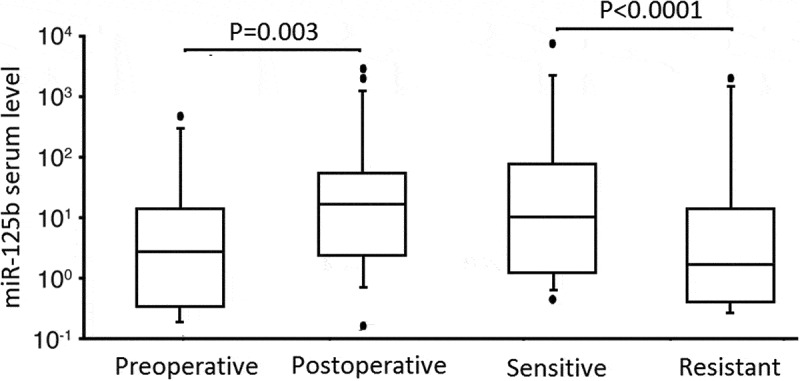
RT-qPCR detection of serum levels of miR-125b in surgical patients and chemotherapy patients.

A statistically signiﬁcant association was observed between preoperative and postoperative serum miR-125b levels in patients with EOC (0.783 ± 0.174 vs 0.916 ± 0.203, P = 0.003, Figure 3). Serum miR-125b levels were also a statistically signiﬁcant difference between the sensitive and resistant groups in patients with EOC (0.924 ± 0.186 vs 0.789 ± 0.1762, P < 0.0001, Figure 3).

### Combination of miR-125b with miR-125b as a tumor marker

The diagnostic efﬁciency of serum miR-125b and CA125 levels in distinguishing patients with EOC from patients without cancer were then assessed. On the basis of the ROC curve, the CA125 cutoff value of 35 U/mL was chosen. By the cutoff value, CA125 displayed 51.6% sensitivity and 89.6% speciﬁcity in diagnosis of EOC (Figure 2(b), P = 0.042). However, combined miR-125b with CA125 displayed 83.6% sensitivity and 95.6% speciﬁcity (Figure 2(c), P < 0.001). Therefore, combining the miR-125b with CA125 could improve diagnostic sensitivity and speciﬁcity.

## Discussion

Ovarian cancer is the fifth leading cause of death for women with cancer worldwide [18]. In more than 70% of cases, it is only diagnosed at an advanced stage. Therefore, the identiﬁcation of new tumor biomarkers could be pivotal in the improvement of patient diagnosis and survival. In this study, serum levels of miR-125b were detected in a large cohort of patients with EOC, noncancerous ovarian lesions, benign ovarian diseases, and healthy controls and to assess its diagnostic value in EOC and treatment responses. It was demonstrated that serum levels of miR-125b were signiﬁcantly lower in patients with EOC than that in controls, and also lower in patients with advanced EOC (stage III–IV) and lymph node metastasis than that in patients with early-stage ovarian cancer (stage I–II) and without lymph node metastasis. Using the median value, the levels of miR-125b had a high sensitivity in diagnosis of ovarian cancer. Furthermore, serum levels of miR-125b showed a statistically signiﬁcant difference between preoperative and postoperative patients in the surgical patient group and between sensitive and resistant groups with regard to response after chemotherapy. By combining miR-125b with CA125, the sensitivity and speciﬁcity were higher in diagnosis of EOC. Thus, the current study demonstrated that serum miR-125b levels are useful as a biomarker to detect EOC and to predict the responses to chemotherapy.

In EOC, Zuberi and colleagues [17] demonstrated that serum levels of miR-125b were higher in most patients with cancer and were associated with cancer development, which could be a potential diagnostic and prognostic marker in clinical practice. More importantly, miR-125b may also have potential as a cancer-speciﬁc serum biomarker for various human cancers [19–21]. Other previous studies showed the enforced miR-125b had a potent antitumor activity *in vitro* and *in vivo* models of therapy against EOC [15,22,23].

In the current study, it was demonstrated that the detection of serum miR-125b could be useful as a biomarker for diagnosis and therapeutic monitoring of responses of patients with EOC. However, presently, a single CA125 used as a biomarker has a limitation for both sensitivity and speciﬁcity. However, after a combination of miR-125b, it reached higher sensitivity and speciﬁcity in diagnosis of EOC. In previous studies, many serum proteins were used as tumor markers for cancer diagnosis, because such tests are generally noninvasive, cost-efﬁcient, and highly reproducible. However, available tumor markers that are measurable are few. The main reason is that these biomarkers have low sensitivity and speciﬁcity. In the current study, serum levels of miR-125b showed a comparable sensitivity to CA125 for EOC diagnosis. When in combination with CA125, miR-125b improved the diagnostic sensitivity. Thus, the combination of CA125 resulted in better sensitivity as tumor markers for EOC. Furthermore, serum levels of miR-125b could predict the responses of patients with EOC to surgery or chemotherapy and observe a statistically signiﬁcant difference between preoperative and postoperative patients in surgical patient groups. These data are novel, and we are not aware of any study so far that replicates these ﬁndings. Our study does have some limitations in that some clinicopathologic data were not enough.

## Conclusion

In the present study, serum miR-125b levels were low in patients with EOC. Furthermore, low serum miR-125b levels were related to high FIGO stage and lymph node metastasis in patients with EOC, suggesting that lower serum miR-125b levels had a poor prognosis in the EOC patients. In addition, serum miR-125b levels were lower in chemoresistant EOC patients and higher in EOC patients after surgery than that in the chemosensitive EOC patients and EOC patients before surgery. More importantly, combined serum miR-125b and serum CA125 improved both sensitivity and speciﬁcity in diagnosis of EOC.

## Research h﻿ighlights

1. Serum miR-125b levels were low in patients with EOC.
2. Low serum miR-125b levels were related to high FIGO stage and lymph node metastasis in patients with EOC.
3. Serum miR-125b levels could predict the responses to chemotherapy in patients with EOC.
4. Serum miR-125b levels before and after surgery could predict surgical effect for patients with EOC.
5. Combined serum miR-125b and serum CA125 improved both sensitivity and speciﬁcity in diagnosis of EOC.
